# Ventilation of Cities

**Published:** 1869-02

**Authors:** 


### VENTILATION OF CITIES.

The most philosophical method of ventilating cities is not to use disinfectants but to carry the gases from our markets, slaughterhouses, sinks, sewers and privies downwards and convey them away under ground as soiled water is. If some of the gentlemen connected with the Board of Health will call at our office 176 Broadway, we will communicate something to them, "for their advantage," and "the rest of mankind."

